# Circulating syndecan-1 is reduced in pregnancies with poor fetal growth and its secretion regulated by matrix metalloproteinases and the mitochondria

**DOI:** 10.1038/s41598-021-96077-1

**Published:** 2021-08-16

**Authors:** Damanpreet Garcha, Susan P. Walker, Teresa M. MacDonald, Jon Hyett, Jessica Jellins, Jenny Myers, Sebastian E. Illanes, Jhy K. Nien, Manuel Schepeler, Emerson Keenan, Carole-Anne Whigham, Ping Cannon, Elizabeth Murray, Tuong-Vi Nguyen, Manju Kandel, Joshua Masci, Ciara Murphy, Tess Cruickshank, Natasha Pritchard, Natalie J. Hannan, Fiona Brownfoot, Alexandra Roddy Mitchell, Anna Middleton, Gabrielle Pell, Georgia P. Wong, Stephen Tong, Tu’uhevaha J. Kaitu’u-Lino

**Affiliations:** 1 The Department of Obstetrics and Gynaecology, Mercy Hospital for Women, University of Melbourne, 163 Studley Road, Heidelberg, VIC 3084 Australia; 2 Mercy Perinatal, Mercy Hospital for Women, 163 Studley Road, Heidelberg, VIC 3084 Australia; 3 Sydney Institute for Women, Children and Their Families, Royal Prince Alfred Hospital, Missenden Road, Camperdown, NSW 2050 Australia; 4 University of Manchester, Manchester Academic Health Science Centre, St Mary’s Hospital, Manchester, M13 OJH UK; 5 Department of Obstetrics and Gynaecology and Laboratory of Reproductive Biology, Faculty of Medicine, Universidad de Los Andes, San Carlos de Apoquindo 2200, Las Condes, Santiago de Chile, Chile; 6 Department of Obstetrics and Gynaecology, Clínica Davila, Recoleta 464, Recoleta, Santiago Chile

**Keywords:** Biomarkers, Diseases, Medical research

## Abstract

Fetal growth restriction is a leading cause of stillbirth that often remains undetected during pregnancy. Identifying novel biomarkers may improve detection of pregnancies at risk. This study aimed to assess syndecan-1 as a biomarker for small for gestational age (SGA) or fetal growth restricted (FGR) pregnancies and determine its molecular regulation. Circulating maternal syndecan-1 was measured in several cohorts; a large prospective cohort collected around 36 weeks’ gestation (n = 1206), a case control study from the Manchester Antenatal Vascular service (285 women sampled at 24–34 weeks’ gestation); two prospective cohorts collected on the day of delivery (36 + 3–41 + 3 weeks’ gestation, n = 562 and n = 405 respectively) and a cohort who delivered for preterm FGR (< 34 weeks). Circulating syndecan-1 was consistently reduced in women destined to deliver growth restricted infants and those delivering for preterm disease. Syndecan-1 secretion was reduced by hypoxia, and its loss impaired proliferation. Matrix metalloproteinases and mitochondrial electron transport chain inhibitors significantly reduced syndecan-1 secretion, an effect that was rescued by coadministration of succinate, a mitochondrial electron transport chain activator. In conclusion, circulating syndecan-1 is reduced among cases of term and preterm growth restriction and has potential for inclusion in multi-marker algorithms to improve detection of poorly grown fetuses.

## Introduction

Small-for-gestational-age (SGA) fetuses form a major proportion of pregnancies with poor perinatal outcome given many have true placental insufficiency and fetal growth restriction (FGR)^1^. Identifying SGA and FGR represents a significant health priority in obstetrics, because small fetuses are at three- to fourfold increased risk of stillbirth^2^. Therefore, improved detection of FGR late in pregnancy would represent a significant clinical advance; at risk fetuses could be closely monitored and delivered before stillbirth occurs^2–4^. When FGR is severe, ultrasound approaches are more effective at detecting its presence^5^. However, when placental hypoxia is less severe, current diagnostic methods to identify the presence of FGR fetuses in utero perform modestly^6,7^.

An option to detect FGR that could be widely accessible is a maternal blood biomarker test.

We have recently reported a study where we screened 22 placental proteins for their association with SGA and FGR^8^. The main finding of that report was a new biomarker of placental insufficiency, Serine Peptidase Inhibitor Kunitz Type 1 (SPINT1). Syndecan-1 was identified as a second potential candidate as circulating levels were significantly reduced at 36 weeks’ gestation in 97 women destined to deliver an SGA infant at term relative to 901 controls^8^. In that report, syndecan-1 was not followed up further and the findings were not validated.

Syndecan-1 is a protein highly expressed in placenta^9,10^. It is a heparan sulfate proteoglycan^11^ that has been implicated in the pathogenesis of inflammatory diseases, cancers and infectious diseases^12–17^. There have been reports suggesting that syndecan-1 may be reduced in the placenta and circulation of patients with preeclampsia (a disease also characterised by placental insufficiency^10,18–22^) but there have been no reports on its association with FGR, except our earlier study^8^.

This study aimed to validate our previous findings through assessment of circulating syndecan-1 concentrations in several different high-risk groups and an unselected cohort. We also aimed to study the mechanisms that regulate syndecan-1 release from placenta.

## Results

### Circulating syndecan-1 is reduced at 36 weeks’ gestation

**Figure 1:**
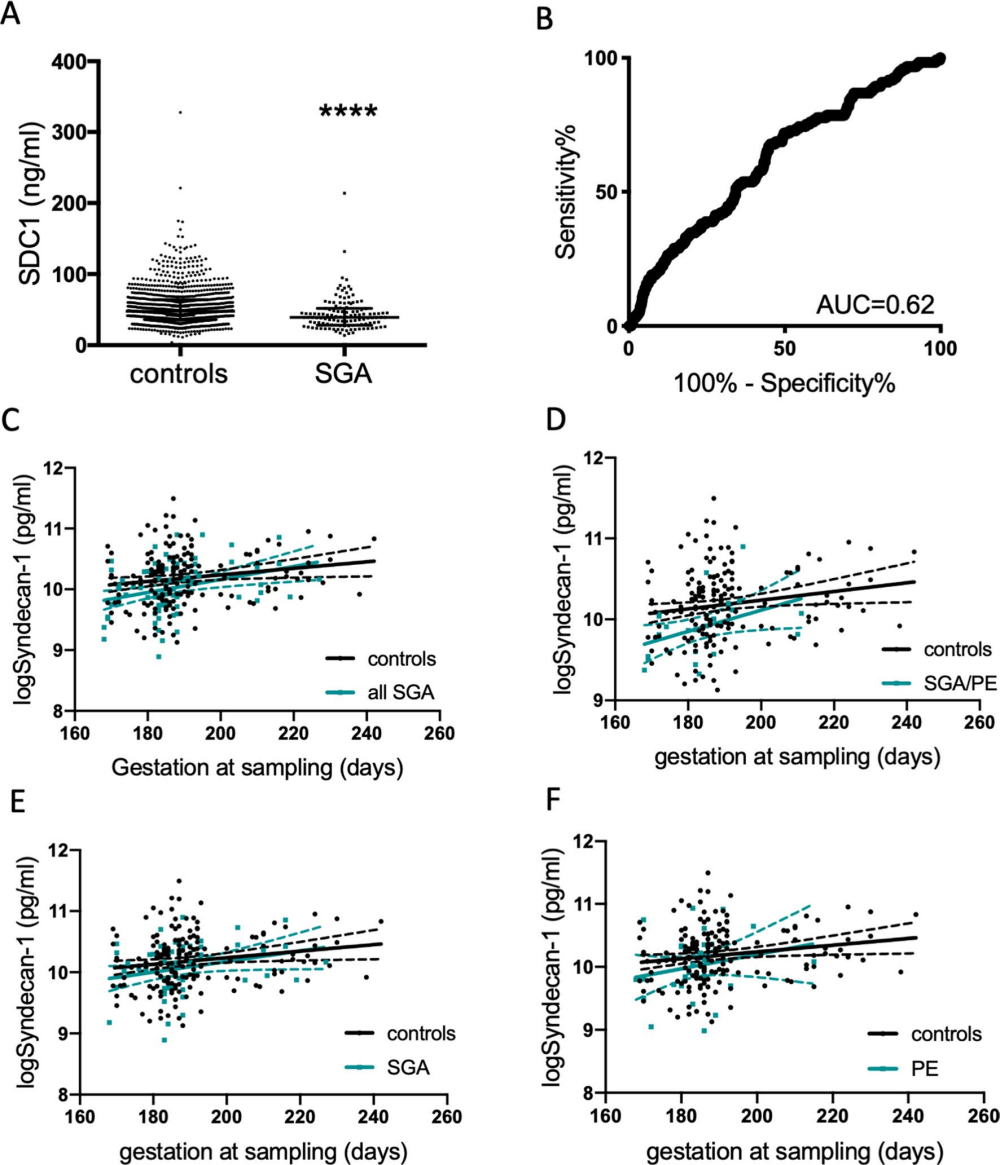
Syndecan-1 is reduced at 36 weeks’ gestation and in women with underlying vascular disease. In samples collected at 36 weeks’ gestation in Sydney Australia (Cohort 1, n = 1085 controls and n = 121 SGA), circulating syndecan-1 was significant reduced (**A**) with an area under the receiver operator curve (AUC) of 0.67 (**B**). Linear regression analyses of logSyndecan-1 in women who attended the Manchester Antenatal Vascular Service (MAViS clinic; Cohort 2) and had plasma sampling across 24–34 weeks’ gestation. (**C**) Controls (n = 171) and women who delivered small-for gestational age (SGA) infants (with or without preeclampsia, n = 82). (**D**) Controls (n = 171) and women who delivered with preeclampsia (PE) and had an SGA infant (SGA/PE n = 25). (**E**) Controls (n = 171) and women who delivered an SGA infant (without preeclampsia, n = 57). (**F**) Controls (n = 171) and women who delivered with preeclampsia and an appropriately grown infant (n = 32). Individual symbols represent a single patient sample. For (**A**), data expressed as median ± IQR, ****p = 0.0001. In panels (**C**–**F**), the solid line is the line of best fit, while the dotted line is the 95% confidence intervals.

We have previously reported that syndecan-1 is reduced at 36 weeks’ gestation^8^. We first set out to validate this finding in a new cohort collected in Sydney, Australia (Cohort 1, Table 1). This cohort comprised samples collected at 36 weeks’ gestation from 1085 controls and 121 women who delivered an SGA baby (birthweight < 10th centile, corrected for gestation). Circulating syndecan-1 levels were significantly reduced (p < 0.0001) in the SGA cohort, with median level of 39.3 ng/ml (Interquartile range (IQR) 28.1–51.7 ng/ml) compared to controls (median of 47.8 ng/ml (IQR 35–64.2 ng/ml); Fig. 1A). The Area under the receiver operator curve (AUC) was 0.62 (Fig. 1B). Thus, this data validates that syndecan-1 is significantly reduced at 36 weeks’ gestation prior to delivery of an SGA infant.

**Table 1:** Maternal characteristics and pregnancy outcomes for Cohort 1, Samples collected at 36 weeks’ gestation in Sydney Australia.

	Controls (n = 1085)	SGA (n = 121)	p value
Age (years)	33.7 (4.2)	33.6 (4.0)	0.56
Booking body mass index (kg/m^2^)	24.7 [22.1–27.9]	25.1 [22.7–28.3]	0.43
Current smoker	15 (1.4%)	6 (5.0%)	0.01
Gestational diabetes	127 (11.7%)	25 (20.7%)	0.009
Nulliparous	637 (58.7%)	83 (68.6%)	0.04
Preeclampsia	19 (1.8%)	6 (5.0%)	0.03
**Mode of delivery**			
Normal vaginal delivery	566 (52.2%)	58 (47.9%)	0.44
Instrumental delivery	210 (19.4%)	25 (20.7%)	
Emergency Caesarean section	149 (13.7%)	14 (11.6%)	
Elective Caesarean section	160 (14.7%)	24 (19.8%)	
Birthweight (g)	3456 [3195–3734]	2687 [2508–2922]	< 0.0001
Birthweight centile	46.6 [28.6–68.3]	5.7 [3.5–8.0]	< 0.0001
Gestational age at delivery (weeks)	39.6 [38.9–40.4]	39.1 [38.0–39.9]	< 0.0001

Data represented as mean (standard deviation) if normally distributed, as median [interquartile range] if not normally distributed, or as number (%) if categorical.

*SGA* small-for-gestational-age (GROW birthweight < 10th centile).

Some percentages do not sum to 100% due to rounding to one decimal place.

### Maternal syndecan-1 is reduced at 24–34 weeks’ gestation in a high risk pregnancy cohort destined to develop SGA

We next measured circulating syndecan-1 in Cohort 2—women attending the Manchester Antenatal Vascular Service (MAViS clinic, in the United Kingdom, Table 2) who have underlying vascular disease (such as chronic hypertension or pre-existing diabetes). Samples were obtained at 24–34 weeks’ gestation.

**Table 2:** Maternal characteristics and pregnancy outcomes for Cohort 2, the Manchester Antenatal Vascular Service (MAViS clinic).

	Controls (n = 171)	SGA (n = 57)	PE (n = 32)	PE/SGA (n = 25)
Age (median) [IQR]	34 [32–38]	35 [30–38.5]	33 [30–36]	35 [31–37]
**Pre-existing hypertension**				
None	27 (15.8)	12 (21)	5 (15.6)	4 (16)
Chronic	115 (67.3)	40 (70.2)	26 (81.2)	18 (72)
Renal	29 (16.9)	5 (8.8)	1 (3.1)	3 (12)
Body mass index (median) [IQR]	30 [25–35]	29 [25.3–33.8]	30 [26–35.8]	30 [25–33]
**Ethnicity (%)**				
Asian	26 (15.4)	6 (10.9)	4 (12.5)	4 (16)
Black	40 (23.7)	17 (30.9)	12 (37.5)	11 (44)
White	86 (50.9)	23 (41.8)	14 (43.8)	7 (28)
Other	17 (10)	9 (16.4)	2 (6.2)	3 (12)
Gestation at sampling (days) (median) [IQR]	187 [182–192]	185 [183–190]	184.5 [180–186.8]	183 [172–190]
Gestation at delivery (days) (median) [IQR]	270 [266–275]	267 [261.5–273]*	259 [245.5–265.8]****	238 [214.5–256]****
Birthweight centile (median) [IQR]	46.4 [28–67.3]	5.7 [3.2–8.2]	39.1 [21.1–61.6]****	2.4 [0.7–5.8]****

Data presented as median [25th–75th percentile], and as number (%) if categorical. Small-for-gestational-age defined as birthweight < 10th centile.*p < 0.05, ****p < 0.0001 compared to control. Ethnicity missing for n = 2 controls, n = 2 SGA.

The MAViS cohort included women who delivered with uncomplicated pregnancies, women who delivered with preeclampsia (and no SGA), women who delivered with preeclampsia with SGA, and women with SGA without preeclampsia. Consistent with the risk profile of the high-risk population who attends the MAViS clinic, rates of these placental complications were higher than expected low risk population. We performed a series of analyses to assess whether syndecan-1 was reduced in these three clinical outcomes (which all represent placental diseases). We adjusted for gestation at sampling and underlying hypertension (Fig. 1C–F). Those who delivered with SGA (with or without preeclampsia, n = 82) had a 0.11 reduction (p = 0.04) in logSyndecan-1 (95% CI − 0.22 to − 0.01) relative to those who had uncomplicated pregnancies (controls, n = 171) (Fig. 1C, Supplementary Table 1). Interestingly, a sub-analysis of the women who had both preeclampsia and SGA (n = 25, suggestive of more significant placental insufficiency) displayed a 0.22 reduction (p = 0.01) in logSyndecan-1 (95% CI − 0.38 to − 0.05) relative to the controls (Fig. 1D, Supplementary Table 2). In contrast, the women who developed SGA without preeclampsia, (n = 57) or indeed women who developed preeclampsia without SGA (n = 32) displayed no significant changes in circulating syndecan-1 (Fig. 1E,F) relative to controls.

Thus, this data demonstrates that circulating syndecan-1 is reduced in women with underlying vascular disease preceding the diagnosis of placental insufficiency (SGA/PE) relative to uncomplicated pregnancies.

### Maternal syndecan-1 is reduced in pregnancies with a small for gestational age fetus

To obtain further evidence that syndecan-1 may be consistently reduced across pregnancy we measured circulating levels in cohort 3, where samples were collected on the day of birth (by caesarean section) in Melbourne, Australia (Supplementary Table 3). Circulating Syndecan-1 concentrations were indeed significantly reduced (p < 0.0001) among those who birthed an SGA infant (n = 53) compared to women who delivered an infant with birthweight > 10th centile (controls, n = 503). The median syndecan-1 concentration in the SGA group was 36.5 ng/ml (IQR 29.1–45.7 ng/ml) compared to 44.3 ng/ml (IQR 35.3–56.9 ng/ml) among controls with an area under the receiver operator characteristic curve (AUC) of 0.67 (Supplementary Figure 1A,B). We next measured circulating syndecan-1 in cohort 4 which also comprised samples collected on the day of caesarean section, but in Santiago, Chile (Supplementary Table 4). In that cohort, we again demonstrated significantly reduced (p < 0.01) circulating levels of syndecan-1: the median syndecan-1 concentration was 39.7 ng/ml (IQR 31.5–46.6 ng/ml) among 46 who birthed an SGA neonate, compared to 45.6 ng/ml (IQR 35.2–57.4 ng/ml) among 359 controls, an AUC of 0.62 (Supplementary Figure 1C,D). Thus, we provide data from two large independent cohorts to demonstrate that circulating syndecan-1 is reduced in women carrying an SGA fetus at term.

### Circulating syndecan-1 is correlated with birthweight centile and placental weight at term

We next assessed whether there was a correlation between circulating syndecan-1 and all birthweight centiles by performing linear regression analyses. This confirmed modest though highly significant associations between circulating syndecan-1 and birthweight at 36 weeks’ (cohort 1; p < 0.0001; R^2^ = 0.02, Supplementary Figure 1E), and on the day of delivery in both the Melbourne (cohort 3; p < 0.0001 R^2^ = 0.06, Supplementary Figure 1F) and Chile cohorts (cohort 4; p = 0.0008, R^2^ = 0.03, Supplementary Figure 1G).

We also considered whether circulating levels were correlated with placental weight. Placental weights were recorded for 96 participants in a Melbourne cohort where blood samples were collected at 36 weeks’ gestation (cohort 5). We demonstrated circulating syndecan-1 at 36 weeks’ gestation was positively correlated with placental weight (p = 0.03, R^2^ = 0.052, Supplementary Figure 1H). Thus, our data demonstrates that circulating syndecan-1 correlates across birthweight centiles, and with placental weight. We also measured plasma syndecan-1 in a small cohort of non-pregnant compared to pregnant (36 week) samples (Supplementary Figure 1I). The median syndecan-1 in the non-pregnant samples was 1634 pg/ml (IQR 256.6–18,389 pg/ml), while the median level in the pregnant cohort was 28,705 pg/ml (IQR 6095–368,366 pg/ml).

### Circulating syndecan-1 in preterm fetal growth restriction

**Figure 2:**
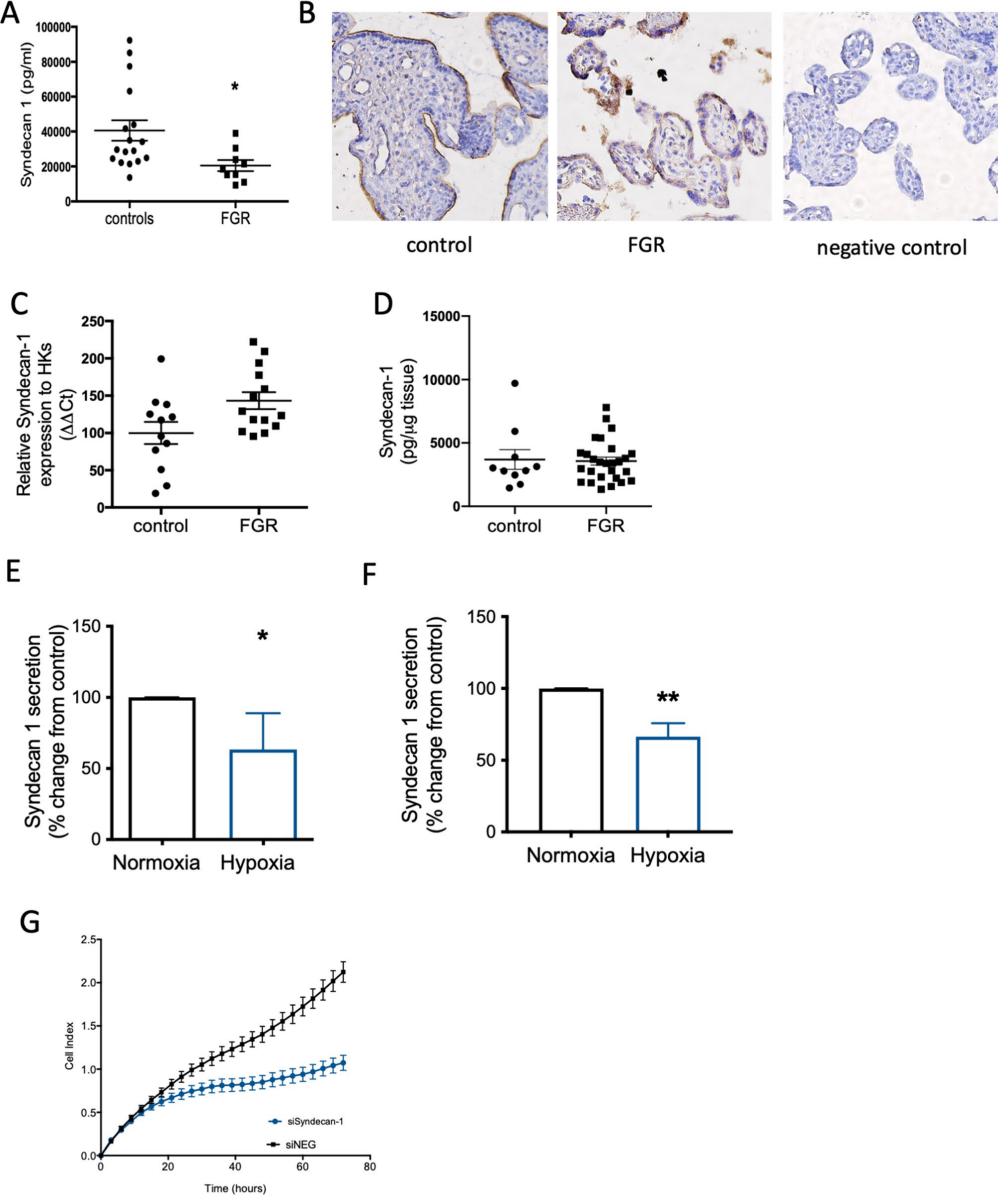
Syndecan-1 is reduced in early onset FGR and by hypoxia and may be important in placental proliferation. Circulating maternal syndecan-1 was measured in a cohort of women who delivered a preterm (< 34 weeks’ gestation—Cohort 5) FGR infant (n = 10) relative to women who delivered healthy infants at term (n = 18 matched for gestation at blood sampling). Circulating syndecan-1 was significantly reduced in the FGR cohort (**A**). Syndecan-1 was localised to the syncytiotrophoblast layer in both pre-term control and FGR placentas (**B**). In placentas obtained from women who delivered a preterm FGR infant (n = 21) relative to preterm controls (n = 12), *syndecan-1* mRNA expression was not significantly changed (**C**). Similarly, no significant difference in protein expression between FGR (n = 27) and preterm control (n = 10) placentas was identified (**D**). When isolated primary cytotrophoblast (**E**) or syncytialised first trimester stem cells (**F**) were exposed to hypoxia (1% O_2_), syndecan-1 secretion was significantly reduced. Finally, in the HTR8/SVneo cell line, when *syndecan-1* was silenced using siRNA, we observed a reduction in cellular proliferation measured using xCELLigence (**G**). Data expressed as mean ± SEM. *p < 0.05, **p < 0.01.

We next measured circulating syndecan-1 in cohort 6, samples collected from women who delivered a preterm (< 34 weeks for fetal indications) FGR infant (< 1st centile birthweight at birth—Supplementary Table 5). Preterm FGR represents a severe variant of the disease where placental dysfunction is advanced, and the stillbirth risk is high. Circulating syndecan-1 levels were significantly reduced (Fig. 2A) in the preterm FGR cohort relative to gestation matched plasma samples from women who delivered a healthy infant at term gestation (> 37 weeks’ gestation) with a normal birthweight.

### Placental syndecan-1 in preterm fetal growth restriction

We next examined whether placental syndecan-1 expression was decreased with preterm FGR. Immunohistochemistry confirmed syndecan-1 localisation to the syncytiotrophoblast layer in preterm control and FGR placentas (Fig. 2B). Interestingly, neither syndecan-1 mRNA (Fig. 2C) or protein expression (measured via ELISA, Fig. 2D) in placentas from women who delivered preterm (< 34 weeks’ gestation) FGR infants (< 1st centile birthweight at birth—Supplementary Tables 6, 7) were reduced compared to gestationally matched placentas (from pregnancies with a fetus of normal birthweight). These placental findings were unexpected given syndecan-1 was consistently reduced in the circulation of pregnancies complicated by FGR. They raise the possibility that molecular regulation of syndecan-1 secretion into the circulation is post translational.

### Primary cytotrophoblast syndecan-1 is reduced under hypoxia and may be involved in regulating placental proliferation

Placental insufficiency is associated with inadequate placental perfusion and hypoxia^23–25^. Therefore, we tested the effects of hypoxia (1% O_2_) or normoxia (8% O_2_) on syndecan-1 expression and secretion. We confirmed reduced syndecan-1 secretion from term primary cytotrophoblast cells (Fig. 2E) and first trimester syncytialised cytotrophoblast stem cells (Fig. 2F) under hypoxic conditions. Silencing syndecan-1 expression in a trophoblast cell line (HTR8/SVneo) using siRNA reduced trophoblast proliferation measured via xCELLigence, where cell growth was continuously monitored over 80 h (Fig. 2G). Together, these data suggest that syndecan-1 secretion may be down-regulated by hypoxia and may play a role in trophoblast proliferation.

### Placental syndecan-1 secretion is regulated by MMPs

**Figure 3:**
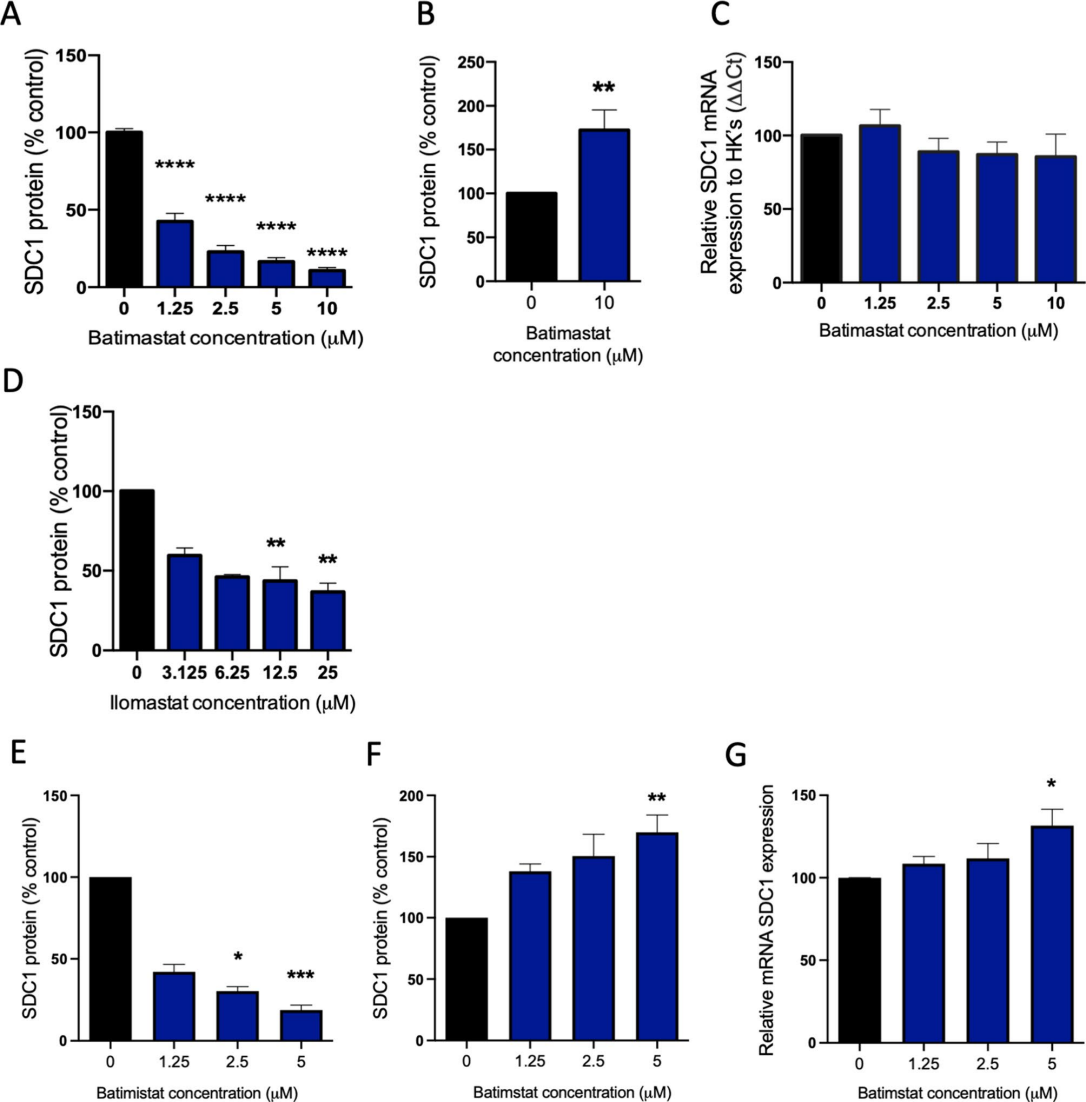
Syndecan-1 is cleaved by matrix metalloproteinases in placental cells. Broad spectrum MMP inhibitor batimastat induced a dose-dependent reduction in syndecan-1 secretion relative to vehicle control (**A**) and caused a significant increase in cellular syndecan-1 (**B**), but no change in *syndecan-1* mRNA expression (**C**). Like batimastat, ilomastat significantly reduced syndecan-1 secretion (**D**). The same effects of batimastat were observed in syncytialised first trimester cytotrophoblast stem cells, with significantly reduced syndecan-1 secretion (**E**), significantly increased cellular protein (**F**) and mRNA expression (**G**). Cellular studies were repeated n = 5 times. Data is expressed as mean ± SEM. *p < 0.05, **p < 0.01, ****p < 0.0001.

MMPs have been shown to cleave syndecan-1 in several human cells and may represent a mechanism of post-translational regulation of its secretion in placenta^26,27^. Treating primary cytotrophoblast with the broad-spectrum MMP inhibitor batimastat, resulted in a significant (p < 0.0001) dose dependent reduction in syndecan-1 secretion (Fig. 3A). Batimastat also significantly (p < 0.01) increased cellular syndecan-1 protein (Fig. 3B) but induced no change in syndecan-1 mRNA expression (Fig. 3C). These findings suggest that MMPs contribute to syndecan-1 release from placenta.

To validate this finding, we treated primary cytotrophoblast with a second broad-spectrum MMP inhibitor, ilomastat. Ilomastat also reduced syndecan-1 secretion in a dose-dependent manner (p < 0.01, Fig. 3D).

Similarly, when we assessed the effect of batimastat on syncytialised cytotrophoblast stem cells^28^, we found a dose dependent reduction in syndecan-1 secretion (Fig. 3E), as well as elevated cellular syndecan-1 protein (Fig. 3F) and mRNA expression (Fig. 3G) at a dose of 5uM. Together these findings suggest that MMPs contribute to syndecan-1 release from placenta.

### Syndecan-1 expression and secretion are regulated via the mitochondrial electron transport chain in placenta

**Figure 4:**
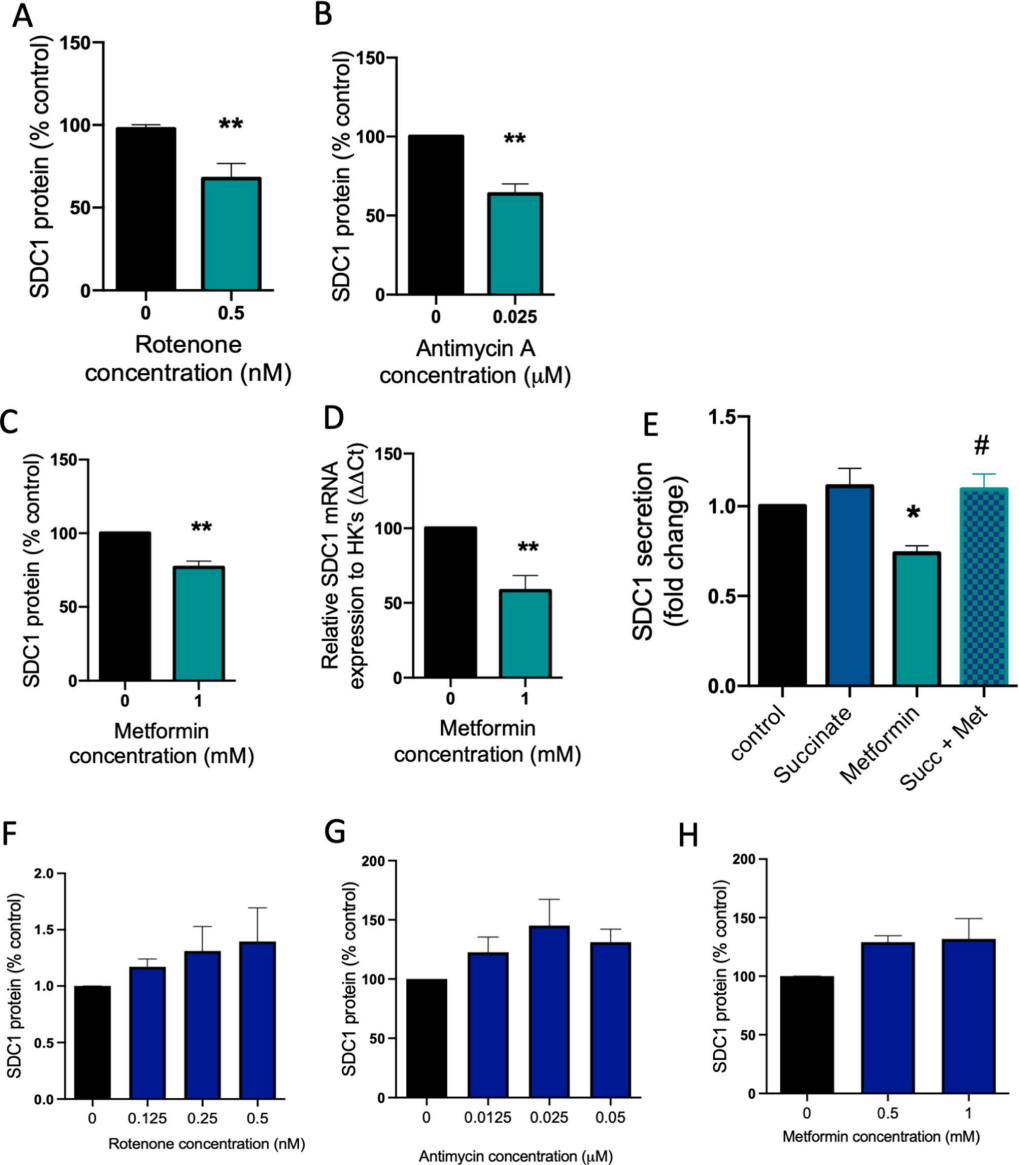
Soluble syndecan-1 may be regulated through the mitochondrial electron transport chain. Isolated primary trophoblast were treated with mitochondrial electron transport chain complex I inhibitor rotenone (**A**) or complex III inhibitor antimycin A (**B**). Low doses of both caused a significant reduction in syndecan-1 secretion. Similarly, when isolated cytotrophoblast were treated with mitochondrial electron transport chain complex I inhibitor metformin, syndecan-1 secretion was significantly reduced (**C**) as well as *syndecan-1* mRNA expression (**D**). Isolated primary cytotrophoblast were also treated with complex I inhibitor metformin in the presence of complex II activator succinate. Whilst metformin significantly reduced syndecan-1 secretion, this effect was reversed when succinate was added (**E**). Contrary to term cytotrophoblast, no effect on syndecan-1 secretion was observed following treatment with rotenone, antimycin or metformin in syncytialised cytotrophoblast stem cells (**F**–**H**). Data is expressed as mean ± SEM. *p < 0.05, **p < 0.01, relative to control, ^#^p < 0.05 relative to metformin treated cells.

Given reports of compromised mitochondrial function in FGR placentas^29,30^, we assessed whether mitochondrial inhibition might alter syndecan-1 secretion. We administered low-dose rotenone (a mitochondrial electron transport chain complex I inhibitor) and Antimycin-A (a mitochondrial electron transport chain complex III inhibitor) and assessed the effects on syndecan-1 secretion. Both inhibitors significantly (p < 0.01) reduced syndecan-1 secretion from primary cytotrophoblast (Fig. 4A,B).

Metformin is also a known mitochondrial electron transport chain complex I inhibitor. In agreement with our findings for rotenone, treating primary trophoblast with metformin significantly reduced (p < 0.01) syndecan-1 secretion (Fig. 4C) and syndecan-1 mRNA expression (Fig. 4D). Thus, our mechanistic studies that used three mitochondrial electron transport chain inhibitors suggest that syndecan-1 secretion is regulated through this organelle.

To further confirm this finding, we next performed a rescue experiment to see whether we could reverse syndecan-1 inhibition by metformin, by activating the mitochondrial electron transport chain downstream of complex I of the mitochondrial electron transport chain. To do this, we treated primary cytotrophoblast with metformin then added succinate which is a complex II activator^31^. Succinate alone did not significantly alter syndecan-1 secretion. However, when we administered metformin and co-treated with succinate, the reduction in soluble syndecan-1 induced by metformin was lost (p < 0.05, Fig. 4E). This data provides further evidence that the mitochondrial electron transport chain plays a role in syndecan-1 secretion from the placenta.

We also tested the effect of rotenone, antimycin and metformin in on syncytialised cytotrophoblast stem cells^28^, however in contrast to our findings in term cytotrophoblast, we observed no effect on syndecan-1 secretion from these cells (Fig. 4F–H).

## Discussion

Examining six independent cohorts from three countries we have robustly validated the hypothesis that syndecan-1 is significantly reduced in the maternal circulation in pregnancies complicated by SGA or FGR. Importantly, in very large cohorts we have found circulating levels are consistently lower across the second half of pregnancy, raising the possibility it may play a role in driving disease pathogenesis. We have also provided evidence that placental secretion of syndecan-1 may be regulated by MMPs via a post translational mechanism. And we show that the mitochondrial electron transport chain also plays a role in its secretion.

Predictive biomarkers for the other common disease of placental insufficiency, preeclampsia, have successfully translated into the clinic^32–36^. In contrast, the performance of biomarkers to predict and detect FGR have been more disappointing. Currently, placental growth factor (PlGF) is the most widely reported and recognised biomarker of placental insufficiency. However, PlGF’s prediction of FGR falls short of clinical utility, either alone or in combination with sFlt-1^37–40^. 36 weeks’ gestation offers a good time for surveillance of fetal growth to prevent late-term stillbirth, given stillbirth rates rise sharply close to term gestations, and intervention after this time point would not inflict significant prematurity on the infant. Sovio and others have recently reported a ratio of metabolites measured at 36 weeks’ gestation improves prediction of FGR over previously reported ratios of sFlt1/PlGF^41,42^. We have recently reported on SPINT1 at 36 weeks as a single biomarker that provides improved sensitivity at predicting SGA compared to PlGF alone^8^. Circulating syndecan-1 was also significantly reduced at 36 weeks’ gestation in women destined to deliver SGA infants at term, and was equivalent to, or potentially out-performed PlGF (AUC 0.63 for syndecan-1 vs 0.59 for PlGF^8^). Here we have validated this finding in five cohorts, indicating that syndecan-1 is consistently reduced in the circulation of women destined to deliver an infant of < 10th centile birthweight. We have also shown linear correlations between syndecan-1 and birthweight centile, both when measured at 36 weeks’ gestation and on the day of delivery. AUCs (ranging from 0.62 at 36 weeks to 0.67 on the day of delivery) suggest syndecan-1 may not be useful as a lone biomarker. However, the fact we found syndecan-1 is consistently decreased with SGA and FGR raises the possibility that it has potential as a clinical biomarker if combined with other circulating biomarkers, or with ultrasound. Syndecan-1 is highly expressed in placenta relative to other human tissues. Our data demonstrating significantly elevated circulating levels in pregnant, vs non-pregnant individuals suggest a pregnancy associated increase that may originate from the placenta. However, we also note that syndecan-1 is expressed in other human organs including the lungs and liver, which may also be contributing sources during pregnancy.

FGR is a leading risk factor for stillbirth, hence our work to identify novel biomarkers for placental insufficiency is clinically relevant. Indeed, a key component of prenatal care is detection of FGR to institute surveillance, and closely monitored, well-timed delivery to avoid stillbirth^40^. Existing tools such as symphysis fundal height, detect only 20% of babies destined to be born small, and even universal ultrasound detects just over half^43^. Accordingly, a placental biomarker, measurable in the maternal circulation that could reliably detect the fetus destined to be born small would transform care.

Although syndecan-1 protein expression was not dysregulated in preterm FGR placentas, our mechanistic data suggests the reduced circulating levels may result from post-translational regulation by MMPs; indeed our in vitro data demonstrated that inhibiting MMP activity reduced syndecan-1 secretion from both syncytialised cytotrophoblast stem cells and term cytotrophoblast. It is possible that placental hypoxia associated with FGR might reduce MMP activity and or activation in placenta, with published evidence supporting this notion in first trimester placental cells^44^. Although we did not measure MMP activity in our cells that were exposed to hypoxia, we observed significantly reduced syndecan-1 secretion from both first trimester and term trophoblast under hypoxic conditions. This is suggestive of a potential link between the reduced plasma syndecan-1 and a role for MMPs in cleaving syndecan-1 in placenta. We also provide evidence to suggest that in term placenta the mitochondrial electron transport chain may be involved in regulation of syndecan-1 secretion.

Although we provide compelling evidence that circulating syndecan-1 is reduced in pregnancies complicated by poor fetal growth, our data does not provide evidence that reduced syndecan-1 is causative of fetal growth restriction. Interestingly, studies in heterozygous syndecan-1 deficient mice have demonstrated that offspring in the heterozygotes are significantly lighter than wild-type pups, a phenomenon that continued into adolescent life^45^. As such, further studies to carefully elucidate how reduced syndecan-1 might contribute to poor placental function and fetal growth restriction are needed.

Placental dysfunction, where the fetus is unable to reach its genetically predetermined growth potential, is likely mediated through a diverse range of pathologic pathways, many of which remain unknown. This study validates syndecan-1 as dysregulated in pregnancies complicated by FGR and provides new understanding around its expression and regulation in placenta. It confirms syndecan-1 as a potential biomarker that could be integrated into a multi-marker test to improve the detection of compromised fetal growth to reduce the burden of preventable stillbirth.

## Materials and methods

### Clinical cohorts for measurement of circulating syndecan-1

Clinical samples were obtained from 6 cohorts. All patients gave written informed consent and collections were conducted in accordance with institutional guidelines and regulations. Cohort 1 was a collection at 36 weeks’ gestation in Sydney, Australia and was approved by the Royal Prince Alfred Hospital ethics committee (HREC/17/RPAH/69). Cohort 2 was a collection between 24–32 weeks’ gestation from the Manchester Antenatal Vascular Service (MAViS) and was approved by the National Research Ethics Service Committee North West 11/NW/0426. Cohort 3 and 4 were collected between 36–42 week’s gestation on the day of delivery in Melbourne, Australia (approved by the Mercy Health Research Ethics Committee, R11/34) or Santiago Chile respectively (approved by Clinica Dávila Ethics Committee, IID100017). Cohort 5 was from samples collected at 36 weeks’ gestation in Melbourne Australia with syndecan-1 levels correlated to placental weight at delivery and was approved by the Mercy Health Research Ethics Committee, R14/12. Finally, cohort 6 was a collection of plasma from patients who delivered at < 34 weeks’ gestation due to FGR and was approved by the Mercy Health Research Ethics Committee, R11/34. Full cohort descriptions are provided in the Supplementary Methods.

#### Outcomes and definitions of cases

Maternal characteristics and pregnancy outcomes were obtained from review of each participant’s medical record, investigation results and hospital database entry, by a clinician blinded to any protein levels.

#### Birthweight centile calculations

GROW Bulk Centile Calculator (v8.0.4, 2019) was used to calculate customised birthweight centiles, enabling adjustment of fetal growth centiles for constitutional characteristics such as maternal height, booking weight, parity, ethnicity, fetal sex and gestational age at delivery. Australian European (AUE) ethnicity was used for cohorts 1, 3, 5 and 6 and Chilean ethnicity (CHL) for cohort 2. Missing maternal height or booking weight values were imputed using the relevant population average as provided by the GROW Bulk Centile Calculator (v8.0.4, 2019). SGA was defined as customised birthweight < 10th centile for term deliveries and preterm FGR defined as a birthweight < 10th centile.

### Observational studies using preterm FGR placental tissues

#### Preterm (< 34 weeks) placental samples

Placental samples were obtained from cases of FGR, and gestation matched controls, delivered by caesarean section at < 34 weeks’ gestation. Preterm control placentas were selected from women who underwent caesarean section for indications not affected by placental insufficiency such as vasa praevia, preterm labour or antepartum haemorrhage, without evidence of infection (histopathological examination of the placentas). Collection of placental samples was approved by the Mercy Health Research Ethics Committee (Ethics Approval Number R11/34) and written informed consent obtained from all participants.

Patient characteristics are outlined in Supplementary Tables 6 and 7.

Placental tissue was obtained and processed immediately following delivery. Maternal and fetal surfaces were removed and the sample washed in ice-cold sterile phosphate-buffered saline (PBS). Samples for protein extraction and RNA extraction were collected in RNA*Later*™ stabilisation solution (Thermo Fisher Scientific). Placenta was also fixed in 10% buffered formalin or 4% Paraformaldehyde for histology.

#### Immunohistochemistry for SDC1 in placental sections

SDC1 was localized by immunohistochemistry in placental tissue collected from either fetal growth restricted or preterm control pregnancies. In brief, paraffin sections (5 µm) were dewaxed in xylene and rehydrated through descending grades of ethanol. Sections underwent antigen retrieval via microwaving using 0.01 mol/l sodium citrate buffer (pH 6.0) for 20 min and then incubated in the hot buffer for a further 20 min. Sections were washed for 10 min in Phosphate-buffered saline pH 7.6 (PBS). Following endogenous peroxidase quenching and blocking of non-specific binding, sections were incubated at 37 °C for 1 h with anti-SDC1 antibody (EPR6454, Abcam, Cambridge, UK) in blocking buffer (DAKO). For isotype controls, primary antibody was substituted with rabbit IgG (SC2027, Santa Cruz, Texas, USA). Staining was visualized using the HRP/DAB Detection IHC Kit (Abcam, Cambridge, UK), and lightly counterstained with Harris hematoxylin (Accustain). Sections were then dehydrated and mounted. Staining was visualized and captured using a Leica microscope and camera.

### In vitro studies

#### Primary human cytotrophoblast isolation

Primary cytotrophoblast were isolated as previously described^46^ from normal term placentas and plated at 80–90% confluency in Dulbecco's Modified Eagle Medium (DMEM) containing 10% fetal calf serum and 1% antibiotic–antimycotic (Thermo Fisher Scientific).

#### Culture of human cytotrophoblast stem cells (hTSCs)

Cytotrophoblast stem cell lines (hTSCs) were imported from the RIKEN BRC through the National BioResource Project of the MEXT/AMED, Japan and cultured according to the publication from Okae and colleagues^28^. Syncytiotrophoblasts were differentiated from cytotrophoblasts as previously described^28^.

#### Exposure of placental cells to hypoxia

Term cytotrophoblasts or syncytialised trophoblast cells were allowed to adhere for 24 h before being maintained in a humidified 37 °C incubator at 8% O_2_ (normoxia) or 1% O_2_ (hypoxia) and 5% CO_2_ for 48 h. Lysates were collected for mRNA expression and media collected for measurement of released syndecan-1.

#### HTR8/SVneo culture and syndecan-1 siRNA

Cells were maintained in RPMI media (Thermo Fisher Scientific) containing 10% fetal calf serum (FCS, Hyclone from GE Health) and 1% Penicillin–Streptomycin. For siRNA experiments, cells were transfected in Opti-MEM medium with combination of Lipofectamine RNAiMAX (Thermo Fisher Scientific) and 40 nM siRNA targeting syndecan-1 (Dharmacon, Lafayette, Colorado, US) or negative control siRNA (Qiagen). Knockdown was confirmed via qRT-PCR.

#### xCELLigence to monitor cellular proliferation

xCELLigence (RTCA Systems) allows the monitoring of cellular behaviour in real time via measuring electrical impedance. We assessed HTR8/SVneo proliferation using xCELLigence following syndecan-1 knockdown with siRNA treatment as described above. 5000 cells were plated per well into the 96 well xCELLigence plate and proliferation assessed for 80 h.

#### Matrix metalloproteinase (MMP) inhibitor treatment of primary cytotrophoblast cells and 1st trimesters syncytialised trophoblast stem cells

To assess the effect of inhibiting MMPs on syndecan-1 secretion, primary cytotrophoblast were allowed to adhere for 24 h before being treated with increasing doses of broad spectrum MMP inhibitors Batimastat (Sigma Aldrich; 1.25–10 μM) and Ilomastat (Sigma Aldrich; 3.25–25 μM) or vehicle control (DMSO) at 37 °C under 8% oxygen for 48 h. For 1st trimester cells, syncytialisation was allowed to take place for 48 h before cells were treated with increasing doses of batimastat (1.25–5 μM) at 37 °C under 8% oxygen for 48 h. Media and cells were collected for RNA or protein extraction and subsequent analysis.

#### Treatment of primary cytotrophoblast cells with Phorbol 12-myristate 13-acetate

To assess whether MMP activation would enhance syndecan-1 secretion^47^, primary cytotrophoblast were allowed to adhere for 24 h before being treated with phorbol 12-myristate 13-acetate (PMA, Sigma Aldrich) at 3.125 or 6.25 μM or vehicle control (DMSO). Cells were incubated at 37 °C under 8% oxygen for 48 h. Media was collected for RNA or protein extraction and subsequent analysis.

#### Treatment of primary cytotrophoblast or syncytialised cytotrophoblast stem cells to examine whether syndecan-1 is regulated through the mitochondrial electron transport pathway

Isolated primary cytotrophoblast were allowed to adhere for 24 h before being treated with vehicle control or 1 mM of metformin (Sigma-Aldrich) for 48 h and the conditioned media and cellular lysates collected for assessment of syndecan-1. Similarly, following 24 h adherence, isolated primary cytotrophoblast were treated with vehicle control (ethanol) or 100 μM of resveratrol (Sigma-Aldrich) for 48 h and the conditioned media and cellular lysates collected for assessment of syndecan-1. Mitochondrial electron transport chain inhibitors rotenone and antimycin A (Sigma-Aldrich) (or vehicle controls) were also added to primary cytotrophoblast for 48 h at 0.5 nM or 0.025 μM respectively and the conditioned media collected for assessment of syndecan-1. For cytotrophoblast stem cells, syncytialisation was allowed to take place for 48 h before cells were treated with rotenone (0–0.5 nM), antimycin (0–0.05 μM) or metformin (0-1 mM) for 48 h and conditioned media collected for assessment of syndecan-1.

To assess whether syndecan-1 secretion could be rescued by activation of complex II of the electron transport chain, primary cytotrophoblast were allowed to adhere for 24 h before being treated with vehicle control, 0.5 mM metformin or 25 μM sodium succinate alone, or metformin + sodium succinate, for 24 h before media was collected for analysis via ELISA.

### End point analyses

#### MTS cell proliferation assay

CellTiter 96^®^ AQueous Non-Radioactive Cell Proliferation Assays (MTS, Promega) were used for the sensitive quantification of cell viability following all treatments according to manufacturer instructions. Only doses that had no negative effects on cells were included for analysis.

#### Quantitative RT-PCR

RNA was extracted from placental samples, primary cytotrophoblast cells and HTR8 cell lines using the Genelute™ Mammalian Total RNA Miniprep Kit (Sigma-Aldrich) as per the manufacturer’s instructions. The conversion of RNA to cDNA was performed using the Applied Biosystems™ High-Capacity cDNA Reverse Transcription Kit, as per the manufacturer’s guidelines. 10 × RT Buffer, 25 × dNTP mix (100 nM), 10 × RT Random Primers, RNase Inhibitor, MultiScribe^®^ Reverse Transcriptase and RNase free water along with the extracted RNA (80–150 ng) were used for the reactions. The iCycler iQ5 (Biorad) ran at 25 °C for 10 min, 37 °C for 60 min, 85 °C for 5 min and held at 4 °C until the samples were collected, they were then stored at − 20 °C until subsequent RT-PCR analysis.

To quantify mRNA expression of the genes of interest, quantitative RT-PCR was performed with the use of Taqman gene expression assays (Life Technologies) for Syndecan-1. All PCRs were performed on the CFX384 (Biorad) and run conditions were as follows: 50 °C for 2 min; 95 °C for 10 min, 95 °C for 15 s, 60 °C for 1 min (40 cycles total). All in vitro data was normalised to YHWAZ, an appropriate house-keeping gene used for internal reference and calibrated against the average Ct of experimental control samples. For placental samples the geometric mean of 2 housekeeping genes, TOP1 and CYC1 was used. All samples of cDNA were run in duplicate and the average Cq was used (given the Cq standard deviation was appropriate). Results were expressed as fold change relative to controls.

#### Measurement of syndecan-1 via ELISA

Circulating (plasma) levels of syndecan-1 were measured using the human Syndecan-1 ELISA kit (Invitrogen). Syndecan-1 levels were measured in conditioned culture media from cellular lysates using the human Syndecan-1 DuoSet ELISA kit (R&D Systems) according to the manufacturer’s instructions. Media were assessed neat. Cellular syndecan-1 was measured in placental protein lysates with all samples diluted in 1% BSA in PBS to achieve a concentration of 5 μg.

### Statistical analysis

Data summarized as mean (SEM), median [25th–75th percentile], and number (%) according to distribution. Hypothesis testing between SGA status used Mann–Whitney rank sum test for continuous and Fisher’s exact test for categorical data. Predictive performance was assessed using area under receiver operating characteristic curve (AUC). All in vitro experiments were repeated a minimum of three times with all cells and tissues for each experiment having been obtained from a different placenta/patient. Furthermore, all experiments were performed in technical triplicate. Data was normalised to controls where appropriate. Data was tested for normal distribution using the Anderson–Darling test, D'Agostino and Pearson test, Shapiro–Wilk test, and Kolmogorov–Smirnov test. Based on normality outcomes data was then statistically tested using a Mann–Whitney *U* test, or Kruskal Wallis with Dunn’s post hoc analysis. All statistical analysis was performed using GraphPad Prism 9.0 (GraphPad Software, Inc.).

## Supplementary Information

Supplementary Information.
