# Supplementary material for: Circulating syndecan-1 is reduced in pregnancies with poor fetal growth and its secretion regulated by matrix metalloproteinases and the mitochondria
Source: Sci Rep. 2021 Aug 16;11:16595. doi: 10.1038/s41598-021-96077-1 (PMC8367987; doi:10.1038/s41598-021-96077-1)
Supplement: Supplementary file 1 — Supplementary Information. [file 41598_2021_96077_MOESM1_ESM.docx]

**Circulating syndecan-1 is reduced in pregnancies with poor fetal growth and its secretion regulated by matrix metalloproteinases and the mitochondria**

**Authors names and affiliations**

Damanpreet Garcha^1^, Susan P Walker^2^, Teresa M MacDonald^2^, Jon Hyett^3^, Jessica Jellins^3^, Jenny Myers^4^, Sebastian E Illanes^5,6^, Jhy K Nien^6^, Manuel Schepeler^6^, Emerson Keenan^1^, Carole-Anne Whigham^1,2^, Ping Cannon^1,2^, Elizabeth Murray^1,2^, Tuong-Vi Nguyen^1,2^, Manju Kandel^1,2^, Joshua Masci^1,2^, Ciara Murphy^1,2^ , Tess Cruickshank^1^, Natasha Pritchard^1,2^, Natalie J Hannan^1,2^, Fiona Brownfoot^1,2^, Alexandra Roddy Mitchell^2^, Anna Middleton^2^, Gabrielle Pell^2^, Georgia P Wong^1,2^, Stephen Tong^1,2^, Tu’uhevaha J Kaitu’u-Lino^1,2^

**Corresponding Author :** Associate Professor Tu’uhevaha Kaitu’u-Lino

Mercy Hospital for Women, Dept of Obstetrics and Gynaecology, University of Melbourne. 163 Studley Road, Heidelberg, Victoria 3084, AUSTRALIA

Telephone: +613 8458 4355

Email: t.klino@unimelb.edu.au

**Supplementary Materials and Methods**

*Cohort 1 – Late pregnancy - Royal Prince Alfred Hospital 36 week collection: Sydney, Australia.* Women with singleton pregnancies attending for a routine scan at 36 weeks (as per hospital policy) were recruited to give a maternal venous blood sample for an observational research project aiming to identify biomarkers for fetal growth restriction and stillbirth. Collection to the ‘*Prediction of Adverse Pregnancy Outcome’* (PAPO) cohort is ongoing and aims to recruit 5,000 women. The study has been approved by the local hospital ethics committee (HREC/17/RPAH/69). Pregnancies involving known fetal structural anomaly were excluded.

The clinical characteristics are shown in Table 1.

*Cohort 2 – High risk pregnancies - Manchester Antenatal Vascular Service: MAViS cohort, UK*

To validate our previous observation that syndecan-1 is associated with placental insufficiency we measured syndecan-1 in plasma samples obtained from a high-risk cohort in the United Kingdom, the Manchester Antenatal Vascular Service (The MAViS clinic). Women gave written informed consent to donate samples for future research studies. The study was approved by the National Research Ethics Service Committee North West 11/NW/0426. Women with current hypertension or a hypertensive disorder in a prior pregnancy are referred to the MAViS research clinic from early pregnancy. Such pregnancies are known to have an elevated risk of preeclampsia, SGA or fetal growth restriction.

The inclusion criteria for women in the MAViS study were: 1. chronic hypertension BP ≥140/90 at ≤20 weeks; 2. chronic hypertension requiring antihypertensive treatment ≤20 weeks; 3. pre-gestational diabetes with evidence of vascular complications (hypertension, nephropathy); 4. history of ischemic heart disease and 5. previous early onset preeclampsia. Clinical characteristics are shown in Table 2.

A case-cohort of 285 participants recruited between October 2011 and December 2016 with a plasma sample obtained between 24-34 weeks and complete outcome data were included in the current study. These 285 participants were selected from an overall cohort of 518 participants and included 171 control women and 114 who either delivered with preeclampsia, SGA or both. The clinical characteristics are shown in Table 2. As women attending the MAViS clinic have underlying vascular disease and were sampled across a range of gestations, we performed linear multivariate regression to determine if syndecan-1 was significantly decreased in SGA cases (<10h birthweight centile), correcting for hypertensive status and gestation at sampling. Using the natural logarithm of the syndecan-1 values (logSyndecan-1), we fitted a series of four multivariate linear regression model with logSyndecan-1 as the dependent variable, and chronic hypertension, renal hypertension, pre-eclampsia and gestation in days and group membership (ie case or control) as independent variables.

*Cohort 3 (Day of delivery at term, Australia): FLAG 2, Australia*

The Fetal Longitudinal Assessment of Growth 2 (FLAG2) study recruited 562 unselected women on the day of elective caesarean section at the Mercy Hospital for Women, Melbourne Australia. Women who were aged over 18 years with a well dated singleton pregnancy, 36^+0^ - 42^+0^ weeks’ gestation were eligible to participate. Exclusion criteria included any suspicion of major fetal anomaly or infection; ruptured membranes; labouring women; those who had undergone cervical ripening or steroid administration before the caesarean section; and those who were positive for hepatitis B, C or HIV. A study blood sample was taken at time of intravenous cannula placement, and birthweight centile was calculated as outlined below. The FLAG2 study was approved by the Mercy Health Research Ethics Committee (Ethics Approval Number R11/34) and written informed consent obtained from all participants. Patient characteristics are shown in Supplementary Table 3.

*Cohort 4 (Day of delivery at term, Chile): FLAG 2, Chile*

To validate our findings in an independent and international cohort, blood samples were also collected at Clinica Dávila, Chile. The inclusion and exclusion criteria were the same as described for the Australian FLAG2 cohort. Plasma was collected from 405 unselected women undergoing elective caesarean section and birthweight centile was calculated as outlined below. Collection of samples as part of the FLAG2 Chile Cohort study was approved by Clinica Dávila Ethics Committee (Ethics Approval Number IID100017) and written informed consent obtained from all participants. Patient characteristics are shown in Supplementary Table 4.

*Cohort 5 (late pregnancy): The Fetal Longitudinal Assessment of Growth (FLAG) study, Australia*

The Fetal Longitudinal Assessment of Growth (FLAG) study was a large prospective study undertaken at the Mercy Hospital for Women in Melbourne, Australia, for which details have previously been reported [8]. For this cohort, we assessed correlations between placental weight of 96 participants and circulating syndecan-1 levels. This study was approved by the Mercy Health Research Ethics Committee (Ethics Approval Number R14/12) and written informed consent was obtained from all participants.

*Cohort 6 (Preterm fetal growth restriction), Australia.*

To assess circulating syndecan-1 levels in early onset FGR, plasma samples were also collected from women with preterm FGR (birthweight <10^th^ centile, and delivered <34 weeks’ gestation for fetal indications[23]. Control blood samples were collected at a matched gestation from women who subsequently delivered appropriate for gestational age (AGA; birthweight ≥10^th^ centile) infants at term. Collection of samples was approved by the Mercy Health Research Ethics Committee (Ethics Approval Number R11/34) and written informed consent obtained from all participants. Patient characteristics are shown in Supplementary Table 5.


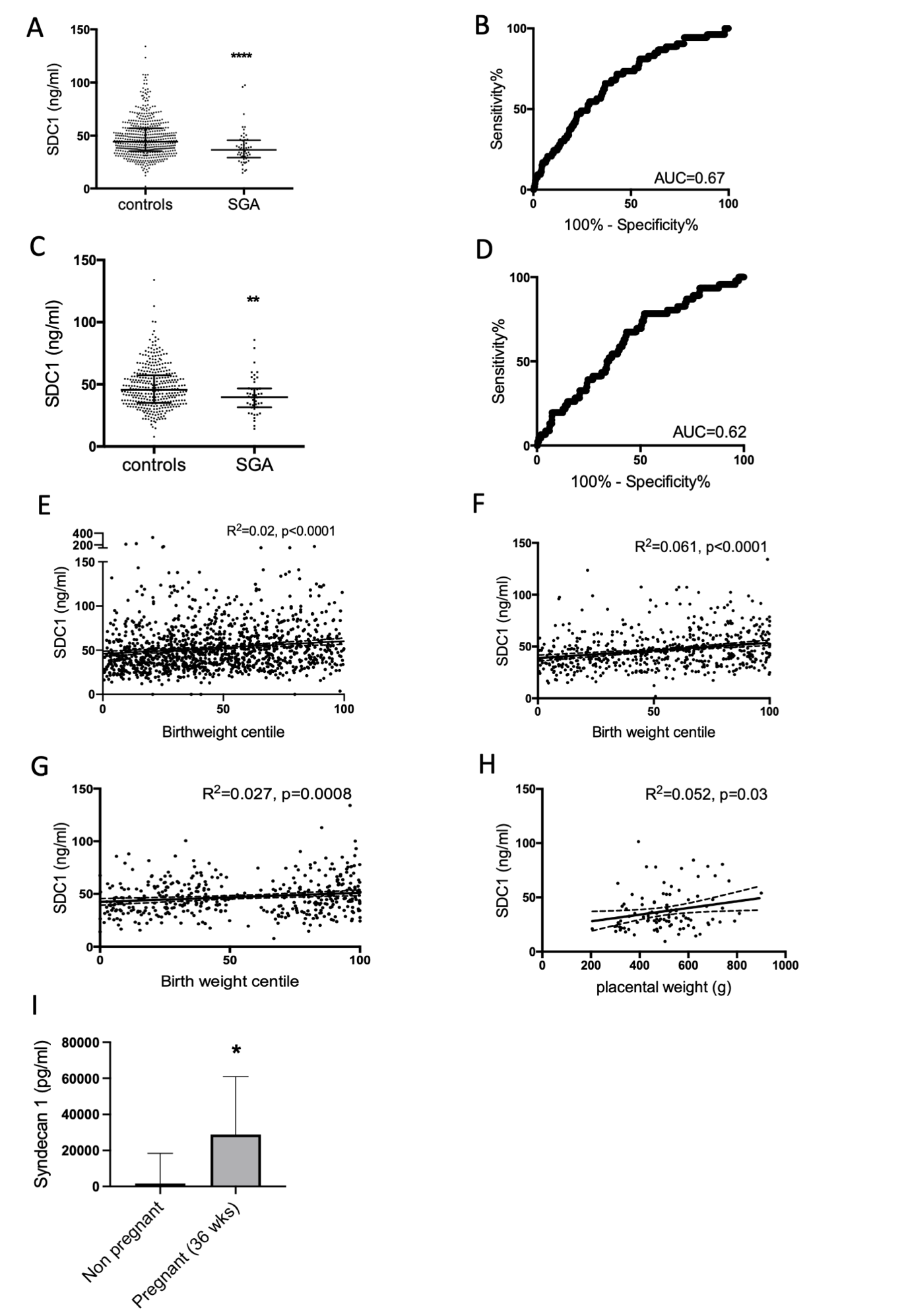


**Supplementary Figure 1: Syndecan-1 is reduced in SGA and correlates with birthweight centile**

Circulating plasma syndecan-1 was measured in 2 cohorts of samples and assessed according to whether the birthweight centile for each baby fell above (controls) or below (SGA) the 10^th^ centile. In samples collected within 1-2 hours of delivery in Cohort 3 (Melbourne, Australia), circulating syndecan-1 levels were significantly reduced in the SGA cohort relative to controls (**A, B;** n=509 controls, n=53 SGA) with an area under the receiver operator curve (AUC) of 0.67. We subsequently measured the levels of syndecan-1 in Cohort 4, a parallel cohort collected in Santiago, Chile (**C, D;** n=359 controls, n=46 SGA). As expected, in this cohort, circulating syndecan-1 was also significantly reduced with an AUC of 0.62. We also assessed the relationship between circulating maternal syndecan-1 and birthweight centile using linear regression analyses in Cohorts 1 (36 weeks, Sydney), 3 and 4 (day of delivery). In Cohort 1 (**E**), Cohort 3 (**F**) and Cohort 4 (**G**), we observed significant associations between the two variables of birthweight centile and circulating syndecan-1. We had also collected placental weights for n=96 samples measured as part of Cohort 5 (sampled at 36 weeks’ gestation). Linear regression demonstrated a significant association between syndecan-1 at 36 weeks’ gestation and placental weight measured on the day of term delivery (**H)**. For comparison of plasma levels in non-pregnant and pregnant individuals, plasma syndecan-1 was measured in non-pregnant participants (n=12) and a small cohort of samples collected at 36 weeks’ gestation (n=12). Levels were significantly higher in the pregnant cohort (**I**). Data expressed as median +/- interquartile range, with each symbol representing a single patient. *p<0.05 **p<0.01, ****p<0.0001

| **Variable** | **Coefficient estimate** | **95% CI** | **p value** |
| --- | --- | --- | --- |
| Chronic hypertension | -0.10 | -0.23 to 0.04 | 0.17 |
| Renal hypertension | 0.38 | 0.20 to 0.55 | <0.0001 |
| SGA with or without pre-eclampsia | -0.11 | -0.22 to -0.01 | 0.04 |
| Gestation at sampling | 0.0069 | 0.0034 to 0.0105 | 0.0002 |

**Supplementary Table 1.** Multivariate linear regression results for the MAViS cohort comparing uncomplicated pregnancies (n=171) to SGA pregnancies (with or without pre-eclampsia ;n=82). Given the wide gestational range at sampling and underlying hypertension, multivariate linear regression with respect to the logSDC1 values was performed to account for gestation at sampling and hypertensive status in the MAViS cohort.

| **Variable** | **Coefficient estimate** | **95% CI** | **p value** |
| --- | --- | --- | --- |
| Chronic hypertension | -0.09 | -0.24 to 0.07 | 0.28 |
| Renal hypertension | 0.40 | 0.21 to 0.60 | <0.0001 |
| SGA with pre-eclampsia | -0.22 | -0.38 to -0.05 | 0.01 |
| Gestation at sampling | 0.0065 | 0.0025 to 0.0105 | 0.0014 |

**Supplementary Table 2.** Multivariate linear regression results for the MAViS cohort comparing uncomplicated pregnancies (n=171) to pregnancies that delivered with both preeclampsia and SGA (n=25). Given the wide gestational range at sampling and underlying hypertension, multivariate linear regression with respect to the logSDC1 values was performed to account for gestation at sampling and hypertensive status in the MAViS cohort*.*

|  | **Controls**  (n=509) | **SGA**  (n=53) | ***P*** |
| --- | --- | --- | --- |
| **Age (mean) [SEM]** | 33.68 [0.2] | 33.86 [0.8] | 0.78 |
| **Booking body mass index (median) [IQR]** | 26.6 [23.3-31.2] | 24.7 [21.9-30.19] | 0.07 |
| **Nulliparous** | 22.2% (113) | 16.7% (9) |  |
| **Cigarette Smoking**   - **Current** - **Non smoker** | 4.5% (23)  95.5% (485) | 7.4% (4)  92.6% (50) |  |
| **Gestation at delivery (weeks) (median) [IQR]** | 38.9 [38.4-39.1] | 38.4 [37.5-38.9] | <0.0001 |
| **Birthweight (g) (median) [IQR]** | 3490 [3180-3748] | 2635 [2435-2815] | <0.0001 |
| **Birthweight centile**  **(median) [IQR]** | 63.4 [35.5-83.2] | 6.3 [2.7-8.0] | <0.0001 |

**Supplementary Table 3:** Maternal characteristics and pregnancy outcomes for Cohort 3 – Samples collected on the day of delivery in Melbourne, Australia.

Data presented as mean (standard deviation) if normally distributed data, as median [25^th^ – 75^th^ percentile] if not normally distributed data, and as number (%) if categorical. Small-for-gestational-age defined as birthweight <10^th^ centile.*p<0.05, **p<0.01, ***p<0.001, ****p<0.0001.

|  | **Controls**  (n=359) | **SGA**  (n=46) | ***P*** |
| --- | --- | --- | --- |
| **Age (Median)** | 31 [28-35] | 31.5 [27-35.25] | 0.9 |
| **Booking body mass index (median) [IQR]** | 26.37 [23.04-32.01] | 27.32 [23.71-29.42] | 0.49 |
| **Nulliparous** | 23% (938) | 26% (12) |  |
| **Cigarette Smoking**   - **Current** - **Non smoker** | 9 (2.5%)   1. (97.5%) | 3 (6.5%)  43 (93.5%) |  |
| **Gestation at delivery (weeks) (median) [IQR]** | 39 [38.4-39.7] | 39.3 [38.5-39.9] | 0.55 |
| **Birthweight (g) (median) [IQR] ****** | 3560 [3300-4890] | 2898 [2691-3265] | <0.0001 |
| **Birthweight centile (Median) [IQR] ****** | 69.6 [32.6-88.7] | 5.65 [3.5-7.9] | <0.0001 |

**Supplementary Table 4:** Maternal characteristics and pregnancy outcomes for Cohort 4 – Samples collected on the day of delivery in Santiago, Chile.

Data presented as mean (standard deviation) if normally distributed data, as median [25^th^ – 75^th^ percentile] if not normally distributed data, and as number (%) if categorical. Small-for-gestational-age defined as birthweight <10^th^ centile. *p<0.05, **p<0.01, ***p<0.001, ****p<0.0001.

|  | **Controls**  **(n=18)** | **Preterm FGR**  **(n=10)** |
| --- | --- | --- |
| **Maternal Age** (years)  Median (IQR) | 32.6 (29.6 – 35.1) | 32.9 (31.0 – 37.4) |
| **Gestation at Delivery** (weeks)  Median (IQR) *** | 39.6 (38.9 – 40.4) | 28.9 (27.9 – 29.7) |
| **Gestation at Blood Collection** (weeks)  Median (IQR) | 28.2 (25.9 – 30.4) | 28.8 (27.5 – 29.1) |
| **BMI** (kg/m^2^)  Median (IQR) | 24.5 (22 – 28.3) | 32.5 (27.3 – 35.9) |
| **Parity** no. (%)  0  1  ≥2 | 5 (27.8)  8 (44.4)  5 (27.8) | 7 (70)  2 (20)  1 (10) |
| **Systolic blood pressure at Delivery** (mmHg)  Median (IQR) *** | 125 (120 – 126) | 170 (147 – 175) |
| **Diastolic blood pressure at Delivery** (mmHg)  Median (IQR) *** | 76 (71 – 80) | 93 (86 – 100) |
| **Birthweight** (g)  Median (IQR) *** | 3415 (3180 – 3678) | 675 (624 – 947) |
| **Birthweight centile**  Median (IQR)**** | 45.1 (28-70.2) | 0 (0-0.1) |
| **Male** no. (%) | 6 (33.3) | 3 (30) |
| **Smoking** no. (%) | 1 (5.6) | 1 (10) |

**Supplementary Table 5:** Maternal characteristics and pregnancy outcomes for Cohort 6 – Plasma samples collected from women delivering FGR infants at <34 weeks’ gestation.

BMI = body mass index, SBP = systolic blood pressure and DBP = diastolic blood pressure. Mann-Whitney U tests used for comparison of medians. Chi-square test used for comparison of categorical variables. *p<0.05 ** p<0.01 ***p<0.001

|  | **Controls**  **(n=12)** | **Preterm FGR**  **(n=21)** |
| --- | --- | --- |
| **Maternal Age** (years)  Median (IQR) | 29.16 (24.4 – 33.6) | 31.6 (23.5– 33.6) |
| **Gestation at Delivery** (weeks)  Median (IQR) | 30 (29.4 - 31.7) | 30.4 (30 – 31.9) |
| **BMI** (kg/m^2^)  Median (IQR) ** | 28.4 (26 – 30) | 25.0 (20 – 30) |
| **Parity** no. (%) **  0  1  ≥2 | 4 (26.7)  6 (40.0)  5 (33.3) | 15 (71.4)  3 (14.3)  3 (14.3) |
| **Systolic blood pressure at Delivery** (mmHg)  Median (IQR) * | 122 (117 – 130) | 135 (117 – 160) |
| **Diastolic blood pressure at Delivery** (mmHg)  Median (IQR) ** | 70 (64 – 76) | 80 (75 – 100) |
| **Birthweight** (g)  Median (IQR) *** | 1587 (1437 – 1876) | 999 (811 – 1237) |
| **Birthweight centile**  Median (IQR)**** | 46.4 (38.2-63.9) | 0.0 (0-0.25) |
| **Male** no. (%) | 5 (41.7) | 11 (61) |
| **Smoking** no. (%) | 1 (6.7) | 2 (11.1) |

**Supplementary Table 6: Placental samples -** maternal characteristics and pregnancy outcomes from women delivering at <34 weeks’ gestation who donated placental samples for mRNA analyses

BMI = body mass index, SBP = systolic blood pressure and DBP = diastolic blood pressure. Mann-Whitney U tests used for comparison of medians. Chi-square test used for comparison of categorical variables. BMI data missing for 4/21 FGR samples and 3/12 controls. *p<0.05 ** p<0.01 ***p<0.001 ****p<0.0001

|  | **Controls (n=10)** | **FGR (n=27)** |
| --- | --- | --- |
| **Maternal Age** (years)  Median (IQR) | 28.9 (22.0 – 34.8) | 31.6 (29.1 – 33.4) |
| **Gestation at Delivery** (weeks)  Median (IQR) | 31.1 (29.5 – 31.9) | 32.2 (25.2 – 33.6) |
| **BMI** (kg/m^2^)  Median (IQR) | 26.2 (23.4 – 34.7) | 26.4 (21.9 – 31.5) |
| **Parity** no. (%) **  0  1  ≥2 | 3 (30)  5 (50)  2 (20) | 20 (74)  3 (11)  4 (15) |
| **Systolic blood pressure at Delivery** (mmHg)  Median (IQR)** | 120 (110 – 127.5) | 135 (120 – 160) |
| **Diastolic blood pressure**  (mmHg)  Median (IQR)* | 74 (70 – 80) | 85 (76.5 – 100) |
| **Birthweight** (g)  Median (IQR)**** | 1738 (1485 – 1873) | 999 (790 – 1232) |
| **Birthweight centile**  Median (IQR)**** | 39.6 (25.6-63.7) | 0 (0-0.3) |
| **Male** no. (%) | 5 (50) | 14 (58.3) |
| **Smoking** no. (%) | 1 (7.7) | 2 (8.3) |

**Supplementary Table 7: Placental samples -** maternal characteristics and pregnancy outcomes from women delivering at <34 weeks’ gestation who donated placental samples for protein analyses

BMI = body mass index, SBP = systolic blood pressure and DBP = diastolic blood pressure. Mann-Whitney U tests used for comparison of medians. Chi-square test used for comparison of categorical variables. BMI data missing for 4/27 IFGR samples and 3/10 controls. *p<0.05 **p<0.01 ****p<0.0001
